# Different healing process of esophageal large mucosal defects by endoscopic mucosal dissection between with and without steroid injection in an animal model

**DOI:** 10.1186/1471-230X-13-72

**Published:** 2013-04-25

**Authors:** Kouichi Nonaka, Mitsuo Miyazawa, Shinichi Ban, Masayasu Aikawa, Naoe Akimoto, Isamu Koyama, Hiroto Kita

**Affiliations:** 1 Department of Gastroenterology, Saitama Medical University International Medical Center, 1397-1 Yamane, Hidaka, 350-1298, Japan; 2 Department of Surgery, Saitama Medical University International Medical Center, Hidaka, 350-1298, Japan; 3 Department of Pathology, Saiseikai Kawaguchi General Hospital, Kawaguchi, 332-8558, Japan

**Keywords:** Esophagus, Stricture, ESD, Steroid, Myofibroblast

## Abstract

**Background:**

Stricture formation is one of the major complications after endoscopic removal of large superficial squamous cell neoplasms of the esophagus, and local steroid injections have been adopted to prevent it. However, fundamental pathological alterations related to them have not been well analyzed so far. The aim of this study was to analyze the time course of the healing process of esophageal large mucosal defects resulting in stricture formation and its modification by local steroid injection, using an animal model.

**Methods:**

Esophageal circumferential mucosal defects were created by endoscopic mucosal dissection (ESD) for four pigs. One pig was sacrificed five minutes after the ESD, and other two pigs were followed-up on endoscopy and sacrificed at the time of one week and three weeks after the ESD, respectively. The remaining one pig was followed-up on endoscopy with five times of local steroid injection and sacrificed at the time of eight weeks after the ESD. The esophageal tissues of all pigs were subjected to pathological analyses.

**Results:**

For the pigs without steroid injection, the esophageal stricture was completed around three weeks after the ESD on both endoscopy and esophagography. Histopathological examination of the esophageal tissues revealed that spindle-shaped α-smooth muscle actin (SMA)-positive myofibroblasts arranged in a parallel fashion and extending horizontally were identified at the ulcer bed one week after the ESD, and increased contributing to formation of the stenotic luminal ridge covered with the regenerated epithelium three weeks after the ESD. The proper muscle layer of the stricture site was thinned with some myocytes which seemingly showed transition to the myofibroblast layer. By contrast, for the pig with steroid injection, esophageal stricture formation was not evident with limited appearance of the spindle-shaped myofibroblasts, instead, appearance of stellate or polygocal SMA-positive stromal cells arranged haphazardly in the persistent granulation tissue of the ulcer site.

**Conclusions:**

Proliferation of spindle-shaped myofibroblasts arranged in a parallel fashion is likely to play an important role in stricture formation after circumferential mucosal defects by esophageal ESD, which may be related to the thinning of the proper muscle layer in the healing course of the defects. Local steroid injection seems to be effective to prevent the stricture through the modification of this process.

## Background

Through the recent development of endoscopy techniques such as iodine staining or magnifying endoscopy with narrow-band imaging (NBI), the number of esophageal squamous cell neoplasms (SCNs) for which local endoscopic treatment is indicated has distinctly increased [1-3]. Endoscopic mucosal resection (EMR) has been widely applied for superficial SCNs as an alternative to surgical therapy, because of the considerable rates of surgical mortality and postsurgical complications related to esophagectomy (range 2.1% to 13.7%), resulting in poor quality-of-life [4-6]. The effectiveness of EMR is underlain by its long-time outcomes that are similar to those of surgical therapy for early-stage esophageal neoplasms[7,8]. In recent years, endoscopic submucosal dissection (ESD) has been developed as a method to resect superficial gastric cancers, which has enabled us to perform precise resection irrespective of the size and shape of the lesions [9,10]. ESD is widely accepted as a reliable therapeutic procedure for superficial esophageal SCNs as well as superficial gastric cancers [11,12].

Esophageal stricture formation is one of the major complications after the endoscopic removal of superficial SCNs. It makes oral food intake difficult and can markedly impair the quality of life. Mizuta et al. studied 47 patients who underwent esophageal ESD, and reported that the mean mucosal defect size by circumferential percentage in seven patients who developed esophageal stricture was 80.4% [13]. Accordingly, it is now widely accepted in Japan that the involvement of less than two-third of the esophageal circumference is one of the criteria to determine whether the neoplasm is suitable for endoscopic treatment, considering the risk of stricture formation after the treatment. Therefore, prevention of the stricture after the treatment can potentially expand the indication of ESD for superficial esophageal SCNs because ESD is technically applicable even for the en bloc resection of the SCNs involving the whole circumferences. In 1969, Holder et al. first reported local corticosteroid injections for the treatment of benign esophageal strictures of dogs and children [14,15], which, during the last decade, has increasingly been used in the treatment of refractory benign esophageal strictures [16-20]. This treatment has recently been adopted to prevent stricture formation after ESD of esophageal superficial SCNs [21,22].

However, while those therapeutic techniques has been developed and used effectively, the fundamental pathological alterations resulting in esophageal stricture formation after large mucosal defects by endoscopic resection and those relating to preventive effects of steroid injection have not been well analyzed so far. Thus, we performed herein an experimental study using pigs, in which we performed esophageal ESD creating circumferential mucosal defects, followed up the postoperative course by endoscopy to observe the process of stricture development, and analyzed their pathological alterations. We also studied modification of the healing process of the circumferential post ESD ulcer by local steroid injection.

## Methods

### Experimental animals and time table

This study was conducted at the animal facility in Saitama Medical University International Medical center, Saitama, Japan, after the approval of Animal Care and Use Committee. Four female domestic pigs with a mean weight of 19 kg (15–21 kg) were used.

Before ESD, the animals were deprived of solid food for 24 hours allowing full access to water. Pre-anesthesia sedation with 2mg/kg ketamine was followed by general anesthesia with sevoflurane, nitrous oxide, and oxygen after endotracheal intubation. For each pig, esophageal ESD was performed creating a circumferential mucosal defect as described below. Postoperative course was followed-up by endoscopy, which was performed at the time of immediately, three days, and every week after the ESD. During the follow-up, changes in food intake were monitored.

**Figure 1:**
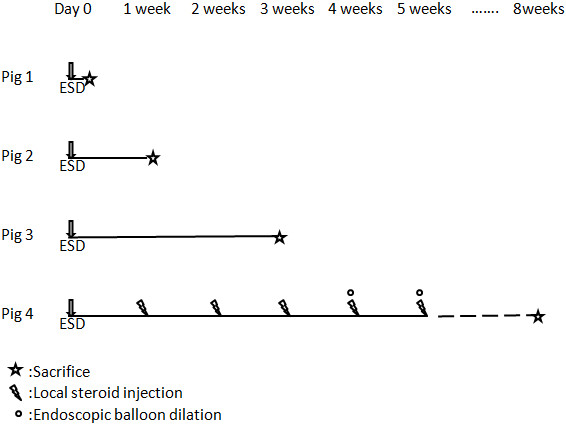
Time table of the experiment.

The three pigs were sacrificed at the time of five minutes, one week, and three weeks after the ESD, respectively (Figure 1). For the remaining one pig, five times of steroid injections into the ulcer beds were performed every week after the ESD until five weeks as described below. This pig was sacrificed at the time of eight weeks after the ESD (Figure 1). The esophageal tissues of the sacrificed pigs were subjected to pathological analysis.

### Endoscopic submucosal dissection

**Figure 2:**
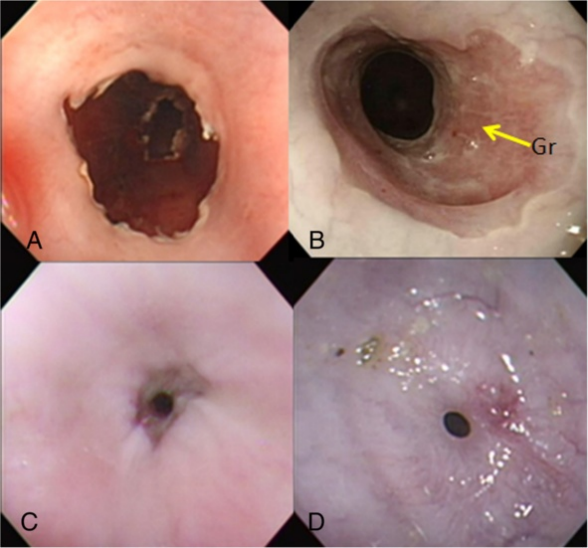
**Endoscopic findings after ESD without steroid injection. (A)** A circumferential mucosal defect immediately after the ESD. **(B)** One week after the ESD, the ulcer bed is mostly covered with granulation tissue. (arrow, Gr: granulation tissue) **(C)** Two weeks after the ESD, the stricture of the ulcer site is remarkable. **(D)** Three weeks after the ESD, the stricture shows a pinhole-like appearance, the surface of which is completely covered with the regenerated epithelium.

ESD and follow-up endoscopy were performed using a GIF-Q240 endoscope (10.2 mm in diameter; Olympus Optical Co, Ltd, Tokyo, Japan) according to the already described procedure[9,23]. Following the injection of a small amount of saline into the esophageal submucosa of the targeted site which was approximately 35 cm from the mouth, 0.5% sodium hyaluronate containing a small amount of epinephrine and indigocarmine dye was injected making a protrusion of the targeted mucosa. With a FlushKnife (KD-2618 JN-15, Fujinon, Tokyo, Japan), the first circumferential linear mcosal incision at the site of 35 cm from the mouth and the second one at the site of 1.5 cm orally from the first incision were made followed by submucosal dissection stating from the first incision, finally creating a circumferential mucosal defect 1.5 cm in width (Figure 2A).

### Endoscopic steroid injection

Triamcinolone acetate (Kenacort ampoule, 40mg/mL, Squibb, Turkey) was diluted with saline and ampoules of 0.5mL of solution containing 10mg triamcinolone acetate were prepared. Each 0.5 mL of solution was gently injected into the shallow part of the ulcer bed at 0, 3, 6, and 9 o’clock sites of the circumferential ulcer using a 5-mm long 25G needle for sclerotherapy.

### Histological analysis

The four esophageal samples obtained from the sacrificed pigs were opened longitudinally and observed grossly. They were fixed in 10% buffered formalin for 24 hours and dissected longitudinally into two to seven pieces at 4 mm intervals (The number of the dissected pieces in the case with a severe stricture was small because it was hard to obtain many sections due to the narrow diameter). Each piece was embedded in a paraffin block, cut in 4μm in thickness, and stained with hematoxylin and eosin (HE). Serial sections were cut for immunohistochemistry of α-smooth muscle actin (SMA) and desmin. Monoclonal mouse anti-SMA prediluted antibody (ASM-1, PROGEN Biotechnik, Heidelberg, Germany) and monoclonal mouse anti-desmin antibody (De-R-11, Dako, Glostrup, Denmark), both at a dilution of 1:100, were used. Antigens were retrieved by heating the tissue sections in 1 mM EDTA solution, pH9.0 for 20 minutes. Immunohistochemical staining was visualized with Envision™ System (Chem Mate Envision/HRP (DAB) kit, Dako, Glostrup, Denmark). Quantitative evaluation on the microscopic slides was performed by measuring with a scale and with imaging analysis software (NIS-Elements D 3.00, Nikon, Tokyo, Japan).

## Results

### Endoscopic findings in relation to time course, food intake, and with/without steroid injection

One pig was sacrificed five minutes after the ESD. The circumferential esophageal ulcers of the remaining three pigs were completely covered with white coats three days after the ESD, and were mostly covered with granulation tissue one week after the ESD (Figure 2B), when one pig without steroid injection was sacrificed. No obvious strictures were observed within one week after the ESD.

**Figure 3:**
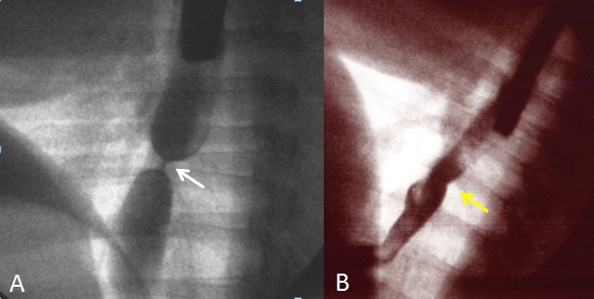
**Esophagographic images after ESD.** (**A**)The image obtained at 3 weeks after the ESD without steroid injection (at the time of the endoscopy shown in Figure 2D). In accord with the endoscopic finding, severe stricture is observed (white arrow). (**B**) The image obtained at 8 weeks after the ESD followed by steroid injection (at the time of the endoscopy shown in Figure 4B). The stricture was significantly milder (yellow arrow) than that shown in (**A**).

Of the remaining two pigs, the pig without steroid injection began to show decreased food intake about 10 days after the ESD, and endoscopy two weeks after the ESD revealed a remarkable esophageal stricture. The GIF-Q240 endoscope could not pass through the stricture (Figure 2C). The food intake of this pig continued decreasing thereafter, and three weeks after the ESD, the stricture became pinhole-like (Figure 2D). The mucosal surface was completely covered with the regenerated epithelium as far as observed on endoscopy. Esophagography also showed severe esophageal stricture (Figure 3A).

**Figure 4:**
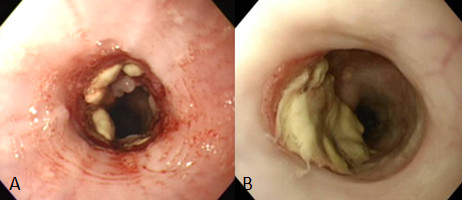
**Endoscopic findings after ESD with steroid injection. (A)** Even three weeks after the ESD, the ulcer bed is covered with white coats. **(B)** Eight weeks after the ESD, the major portion of the ulcer is covered with the regenerated epithelium, but the open ulcer portion is persistent and the stricture is not evident.

For the pig with steroid injection, the ulcer bed was still covered with the white coat two weeks and even three weeks after the ESD (Figure 4A), and the GIF-Q240 endoscope could readily pass through the site of ulcer. Four weeks after the ESD, a slight narrowing at the ulcer site was identified, causing a weak resistance when the endoscope passed through the site. Therefore, after local steroid injection, dilatation was performed at 2 atm for 10 sec at 4 and 5 weeks after the ESD, using a balloon which dilates a stricture to 12 mm at 3 atm and to 15mm at max 8 atm.

On endoscopy eight weeks after the ESD when this pig was sacrificed, the regenerated epithelium covered the major portion of the ulcer with the white coat remained in less than half of the lesion (Figure 4B). The endoscope could readily pass through the site of the ESD. Esophagography revealed that stenotic change of the ESD site was very mild (Figure 3B). No decrease in food intake was noted throughout the observation period for this pig.

### Pathological findings in relation to time course and with/without steroid injection

**Figure 5:**
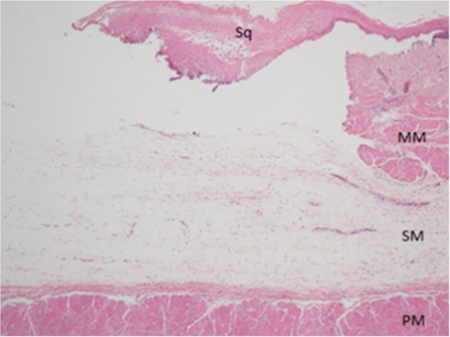
**Histologic findings of the esophageal ulcer five minutes after the ESD.** A sufficient volume of the submucosal layer remains in the ulcer bed with no evidence of damage to the proper muscle (striated muscle) layer. Neither inflammatory reaction nor reparative change is noted. Sq: squamous epithelium, MM: muscularis mucosa, SM: submucosa, PM: proper muscle layer (These symbols are used in the same way in the figures below). HE, original magnification × 40.

Gross examination of the ulcer bed of the esophagus five minutes after the ESD showed a sufficient amount of hyaluronic acid remained in the submucosa. Histological examination also showed a sufficient volume of the submucosal layer remained with no evidence of damage to the proper muscle (striated muscle) layer (Figure 5). Neither inflammatory reaction nor reparative change was noted. Immunohistochemically, desmin was positive for the muscularis mucosa, proper muscle fibers, and the media of some muscular blood vessels whereas SMA was positive for the muscularis mucosa, some scattered proper muscle fibers, the media of the muscular blood vessels, and the walls of minute blood vessels and capillaries. No fibroblastic cells positive for desmin or SMA were found.

**Figure 6:**
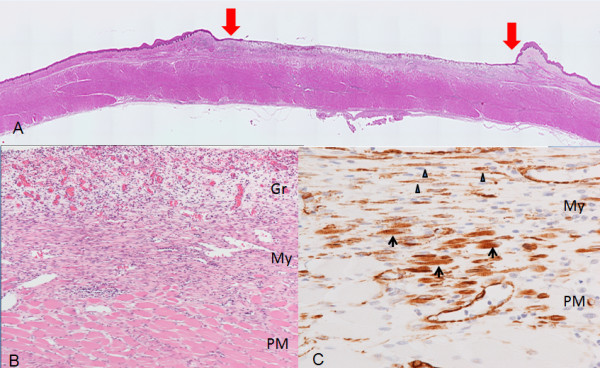
**Histologic findings of the esophageal ulcer one week after the ESD. (A)** The whole mount view of a HE preparation. The regenerative squamous epithelium is observed at the ulcer edges (arrows). The thickness of the proper muscle layer is still preserved. **(B)** In middle power view of the ulcer bed, myofibroblastic cells (My) are identified arranged in a parallel fashion and extending horizontally forming bundles between the edematous granulation tissue of the ulcer bed surface (Gr) and the proper muscle layer (PM), partly occupying the superficial part of the PM. HE, original magnification ×100. **(C)** High power view of the area including the upper proper muscle layer and the lower myofibroblastic cell layer in the section stained for SMA. Transition between SMA-positive spindle-shaped myofibroblastic cells (arrowheads) and SMA-positive small striated myocytes (arrows) is suggested. Immunostain for SMA, original magnification ×400.

The esophagus resected one week after the ESD showed no stricture as was observed on endoscopy. Histologically, the surface side of the submucosaal layer of the ulcer bed was entirely occupied by a layer of edematous inflammatory granulation tissue (Figures 6A and B), and the regenerative squamous epithelium on the granulation tissue was observed at the ulcer edges. No deformity of the outer contour of the proper muscle layer was noted. Between the granulation tissue and the proper muscle layer, layers composed of fibroblastic cells with spindle-shaped nuclei and eosinophilic cytoplasm were formed. These cells were arranged in a parallel fashion and extending horizontally (Figure 6B). These fibroblastic cells were immunoreactive for SMA but negative for desmin, implying that they were myofibroblasts (Figure 6C). These cells partly occupied the superficial part of the proper muscle layer (Figure 6B), and transition between some SMA-positive myocytes and the myofibroblastic cells were suggested (Figure 6C). At the periphery of the ulcer, the myofibroblastic cell bundles were in the same line with the muscularis mucosa, suggesting transition between them, too.

**Figure 7:**
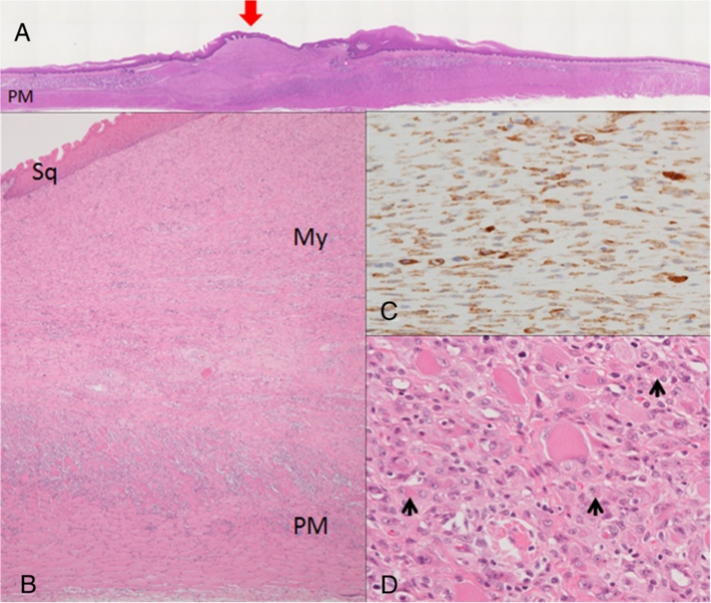
**Histologic findings of the esophageal ulcer three weeks after the ESD. (A)** The whole mount view of a HE preparation showing formation of a luminal ridge covered with the regenerated epithelium (arrow) without deformity of the outer contour of the proper muscle layer. The proper muscle layer of the luminal ridge area is thinned. **(B)** Low power view of the luminal ridge occupied by the thick layer of myofibroblastic cells (My) involving the inner layer of the proper muscle (PM). The ridge surface is covered with the regeneratied squamous epithelium (Sq). HE, original magnification ×40. **(C)** High power view of the myofibroblastic cells in the luminal ridge in the section stained for SMA. SMA-positive myofibroblastic cells, nuclei of which are plumper than those depicted in Figure 6C, are arranged in a parallel fashion and extending horizontally. Immunostain for SMA, original magnification ×400. **(D)** High power view of the proper muscle layer at the thinned portion, in which small immature myocytes are observed (arrows). HE, original magnification ×400.

The esophagus resected three weeks after the ESD, when a complete stricture was noted endoscopically, showed a circumferential hard luminal ridge without deformity of the outer contour of the proper muscle layer. Histologically, the ulcer was healed and the luminal ridge was completely covered with the regenerated squamous epithelium (Figures 7A and B). However, the muscularis mucosa was not regenerated and showed the defect. The ridge was occupied by a thick layer of SMA-positive myofibroblastic cells mostly arranged in a parallel fashion and extending horizontally (Figures 7B and C). The nuclei of these myofibroblastic cells were more swollen than those observed in the esophagus one week after the ESD. In the superficial portion beneath the regenerative epithelium, some myofibroblastic cells were positive for desmin as well as SMA whereas the deeper portion showed more fibrotic appearance with decreasing number of SMA-positive myofibroblastic cells. The proper muscle layer of the ridge portion was thinned by the loss of the inner muscle layer (Figures 7A and B). In the remaining inner muscle layer, immature-appearing myocytes immunoreactive for SMA were observed (Figure 7D).

**Figure 8:**
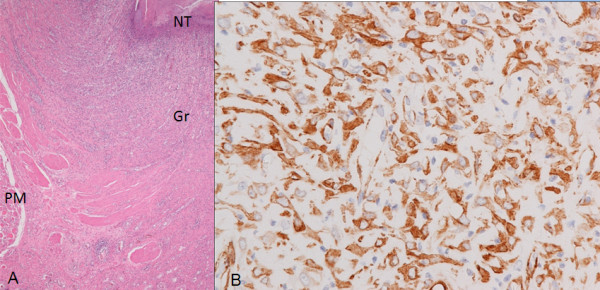
**Histologic findings of the esophageal ulcer with steroid injection eight weeks after the ESD. (A)** Low power view showing the area of the persistent open ulcer. The esophageal wall of the ulcer portion is occupied by transmural inflammatory granulation tissue (Gr) with disruption of the proper muscle layer (PM) and inflammatory necrotic tissue (NT) on the ulcer bed surface. HE, original magnification ×40. **(B)** High power view of the upper half area of the granulation tissue in the section stained for SMA, in which SMA-positive stromal cells appear stellate or polyogonal in shape and are arranged haphazardly. Immunostain for SMA, original magnification ×400.

The esophageal ulcer of the pig that received repeated local steroid injection after the ESD did not heal completely even two months after the ESD, when no obvious stricture was noted on both endoscopy and esophagography. The ulcer portion was occupied by transmural inflammatory granulation tissue with disruption of the proper muscle layer and inflammatory necrotic tissue on the ulcer bed (Figure 8A). The upper half of the granulation tissue contained many SMA-positive desmin-negative stromal cells, but they were stellate or polygonal rather than spindle in shape and arranged in various directions (Figure 8B), being morphologically very different from the above described spindle-shaped myofibroblastic cells. Very limited appearance of bundles composed of spindle-shaped SMA-positive myofibroblastic cells arranged in a parallel fashion and extending horizontally was observed around the granulation tissue. The deeper portion of the granulation tissue was more fibrotic.

### Quantitative pathological findings

Quantitative pathological findings in relation to time course are summarized in Table 1. “n” in Table 1 and below means the number of preparations that were made from the esophagus of each pig as described above in Histological analysis of Materials and Method.

**Table 1:** Quantiative pathological findings in relation to the time course

	**Steroid injection**			**Steroid injection**
	**(−)**			**(+)**
	**Immediately after**	**1 week after**	**3 weeks after**	**8 weeks after**
	**ESD**	**ESD**	**ESD**	**ESD**
	**(n=5)**	**(n=7)**	**(n=2)**	**(n=5)**
Ulcer size evaluated as distance between desmin-positive muscularis mucosa edges (cm, mean±SD)	1.38±0.22	1.54±0.23	0.80±0.14	0.78±0.11
Largest thickness of SMA-positive myofibroblastic cell bundles (μm, mean±SD)	0*	297±70.5	1,156±283	341±216
Thickness of proper muscle layer (μm, mean±SD)	1,231±112	1,444±123	584±218	NA**

SMA: α-smooth muscle actin; SD: standard deviation; NA: not applicable.

“n” means the number of preparations that were made from the esophagus of each pig.

*no SMA-positive myofibroblastic cells identified.

**The muscular layer was disrupted, which made the measurement difficult.

The ulcer size evaluated on the microscopic slides as the distance between the desmin-positive muscularis mucosa edges was 1.38 ± 0.22 cm (mean ± standard deviation, n=5) five minutes after the ESD, 1.54 ± 0.23 cm (n=7) one week after the ESD, 0.80 ± 0.14 cm (n=2) three weeks after the ESD, and 0.78 ± 0.11 cm (n=5) eight weeks after the ESD (the pig with steroid injection).

The largest thickness of the SMA-positive myofibroblastic cell bundles was 297 ± 70.5 μm (mean ± standard deviation, n=7) one week after the ESD, 1,156 ± 283 μm (n=2) three weeks after the ESD, and 341 ± 216 μm (n=5) eight weeks after the ESD (the pig with steroid injection).

The thickness of the proper muscle layer was also measured in the area between the desmin-positive muscularis mucosa edges, and the smallest value was adopted as the thickness. It was 1,231 ± 112 μm (mean ± standard deviation, n=5) five minutes after the ESD, 1,444 ± 123 μm (n=7) one week after the ESD, and 584 ± 218 μm (n=2) three weeks after the ESD. With regard to the pig with steroid injection, the muscular layer was disrupted with granulation tissue at 8 weeks after the ESD, and it was difficult to measure its thickness correctly.

## Discussion

In our pig model, the esophageal stenotic process after a circumferential mucosal defect by ESD began more than one week after the ESD, probably around 10 days after the ESD, and a pinhole-like stricture was completed around three weeks after the ESD. This time course is mostly consistent with the results of some previous experimental studies using pigs or dogs, in which esophageal strictures developed at the time of two to four weeks after creating large mucosal defects [24-26], as is also the case for ESD for human esophagus [13,27]. Therefore, our pig model is considered to be appropriate for studying the healing process of post ESD ulcers of the esophagus.

In the present study, two important pathological features were identified during the healing process of esophageal ulcers after circumferential ESD without steroid injection. One is the appearance of myofibroblasts showing spindle cells arranged in a parallel fashion and extending horizontally, and the other is the loss of the proper muscle layer. As for the former, the layers of myofibroblasts appeared one week after the ESD and increased in thickness three weeks after the ESD when a complete stricture was observed. In contrast, the appearance of such myofibroblasts was very limited in the case with local steroid injection, in which no apparent stricture was noted and SMA-positive stellate or polygonal cells arranged in random directions were observed in the granulation tissue. Thus, it seems to be plausible that the spindle-shaped myofibroblasts forming layers by regular horizontal arrangements play an active role in the stricture formation. Although both ulcers with and without steroid injection shrunk as revealed by measuring the distance between the desmin-positive muscularis mucosa edges, only the ulcer without steroid injection showed a pinhole-like stricture, the mechanism of which must be studied further considering the shape and arrangement of the proliferated stromal cells in the process of ulcer healing. Anyway, the formation of benign strictures of the esophagus has generally been believed to be caused by the production and deposition of collagen fibers stimulated by deep esophageal ulceration and/or chronic inflammation [28]. However, considering the contraction ability of myofibroblasts as well as extracellular matrices production [29], the stricture would not be caused only by the deposition of collagen fibers.

In our experiment without steroid injection, the thickness of the proper muscle layer decreased with loss of the muscle fibers of the inner muscle layer during the healing process of the ESD ulcer resulting in stricture formation. Honda et al. [26,30] stressed destruction and fibrosis of the proper muscle layer during the process of the ulcer healing and/or constriction formation in their experiments with dogs. They speculated that myenteric nerve plexus damage or direct electric damage to the muscle layer might induce atrophy of the muscle fibers. However, based on our observations, we prefer to hypothesize more dynamic alterations rather than passive atrophy of the muscle fibers. In parts of the thinned muscle layer adjacent to the proliferating myofibroblast layer, the myocytes appeared to show a transition to the myofibroblast layer, suggesting that dedifferentiation of myocytes may be involved in the appearance of the myofibroblast layer during the healing process. Whether similar situations occur in the human esophagus should be confirmed in the further study because the proper muscle layer of the human esophagus consists of the smooth muscle except in the cervical portion while that of the porcine esophagus consists of the striated muscle. However, dedifferentiation of muscle cells to myofibroblasts has been reported for both smooth muscle cells and striated muscle cells in various pathological settings. Studies on bronchial smooth muscle cells in patients with bronchial asthma have led to reveal their dedifferentiation into myofibroblasts [31-33]. Recently, Suekane et al. reported the proliferation and migration of moderately differentiated intestinal smooth muscle cells from the muscular layers in Crohn’s disease [34]. As for the striated muscle, Foster et al. demonstrated induction of the dedifferentiation of myocytes to myofibroblastic cells in injured skeletal muscle [35]. Besides, that myofibroblasts dedifferentiated from smooth muscle cells are reportedly involved in the stricture formation of the bronchioles in asthma and that of the intestine in Crohn’s disease is in agreement with our hypothesis, the critical role of myofibroblasts dedifferentiated from the muscle layer on the stricture formation [31-34]. With regard to the sources of myofibroblasts or myofibroblastic cells, bone marrow-derived circulating fibrocytes should also be considered as well as local resident cells such as fibroblasts and muscle cells [36,37].

The present study confirmed the therapeutic potential of steroid injection to prevent esophageal strictures after circumferential ESD. From the results of our study, one of the possible mechanisms would be that steroid injection modifies the appearance and proliferation of the spindle-shaped myofibroblastic cells showing regular horizontal arrangement in the ulcer healing process. However, the detail mechanisms are unclear, and further studies are warranted, in which TGF-β seems to be an important factor to be focused [35].

In the previous reports of clinical use of steroid injection for the management of benign esophageal strictures, most investigators have used triamcinolone acetate [17-20]. Thus, in this study, we also used triamcinolone acetonide with the dose and injection interval according to the report of Kochhar and Marharia [17]. However, it remains to be determined what the optimal injection technique and frequency is, and at what dose triamcinolone should be injected, requiring further studies. Besides, as has been reported [14,17-20], it often seems to be impossible to avoid esophageal stricture by local steroid injection alone. Also in this study, gentle balloon dilatation was required for the steroid injection pig. However, in the current clinical setting, frequent balloon dilatation is performed over several months to avoid stricture formation after esophageal ESD. Whether local steroid injection could be effective to reduce the frequency of balloon dilatation should be confirmed further.

## Conclusions

In conclusion, our study would provide an additional hint to understand the mechanism of stricture formation after circumferential ESD of the esophagus, and to establish therapeutic approach to prevent stricture formation.

## Abbreviations

ESD: Endoscopic submucosal dissection; EMR: Endoscopic mucosal resection; SCN: Squamous cell neoplasm; NBI: Narrow band imaging; HE: Hematoxylin and eosin.

## Competing interests

The authors declare that they have no competing interests.

## Authors’ contributions

KN designed and performed this study, and prepared and submitted the report. MA and MM designed the study, surgically excised the esophagus, and cooperated for the experiment. SB performed pathological analysis. NA prepared samples for the pathological investigation. IK participated in the design of the study and its coordination. HK checked the final version of the manuscript and gave final permission to submit the manuscript. All authors have read and approved the final manuscript.

## Pre-publication history

The pre-publication history for this paper can be accessed here:

http://www.biomedcentral.com/1471-230X/13/72/prepub
